# Idiopathic Spontaneous Intraperitoneal Haemorrhage (ISIH): A Diagnostic Dilemma and Its Conservative Management

**DOI:** 10.7759/cureus.44879

**Published:** 2023-09-08

**Authors:** Katie McComb, Mohammed Barghash, Saleh Eltayef

**Affiliations:** 1 General Surgery, North Manchester General Hospital, Manchester, GBR; 2 General and Colorectal Surgery, North Manchester General Hospital, Manchester, GBR

**Keywords:** pain, abdominal, haemorrhage, intraperitoneal, spontaneous

## Abstract

Idiopathic spontaneous intraperitoneal haemorrhage (ISIH) is a rare cause of acute abdominal pain. It refers to haemoperitoneum resulting from the rupture of an intra-abdominal vessel without the preceding trauma or underlying pathology. Here, we present the case of a 17-year-old boy with acute abdominal pain. Initially, acute appendicitis was the primary differential diagnosis. Imaging demonstrated a significant volume of intra-abdominal fluid that was haemorrhagic in nature, but no active bleeding or source was identified. A conservative management approach was adopted due to the patient’s clinical improvement during his admission. This case highlights the high index of clinical suspicion required to diagnose and investigate ISIH. In contrast to the historical opinion that stipulates management with a surgical intervention, this case demonstrates the possibility of conservative management in stable patients.

## Introduction

Intra-abdominal haemorrhage is usually attributable to a ruptured aortic aneurysm, following a vascular injury (traumatic or iatrogenic), secondary to autoimmune conditions, malignancy or as a consequence of systemic coagulopathy [1,2]. Idiopathic spontaneous intraperitoneal haemorrhage or ISIH is the term given to atypical and atraumatic cases with spontaneous rupture of an intrabdominal vessel [2,3]. ISIH is defined as haemoperitoneum secondary to the tear of an intra-abdominal visceral vessel, in the absence of trauma or underlying pathology, the cause of which cannot be easily identified [1,2].

ISIH is a rare condition with an unknown incidence [2]. It poses a diagnostic challenge as the source of bleeding may not be identifiable through imaging or at the operative intervention. A high index of clinical suspicion along with close monitoring of the patient is required. Although surgical intervention has historically been required in such cases, we present a case of ISIH successfully managed with a conservative approach.

## Case presentation

A 17-year-old boy presented to our institution with a one-day history of abdominal pain. He reported that the pain was initially located in the epigastrium but had subsequently migrated to the right iliac fossa. The pain was associated with a loss of appetite and dizziness. He denied any history of vomiting, change in bowel habit or urinary symptoms. He described difficulty walking upright. His past medical history was significant for Raynaud’s disease and attention-deficit/hyperactivity disorder. His medications included methylphenidate hydrochloride twice per day.

On clinical examination, there was tenderness in the right iliac fossa. However, there was no guarding or rebound tenderness. He was haemodynamically stable and was normotensive at assessment. Haematological and biochemical tests revealed a normal haemoglobin (Hb) level of 122 g/dL, a white cell count (WCC) of 18.3 x 10^9^/L, a platelet count of 252 x 10^9^/L and a C-reactive protein (CRP) level of 2 mg/dL. Clinically, there was a concern of potential appendicitis.

**Figure 1:**
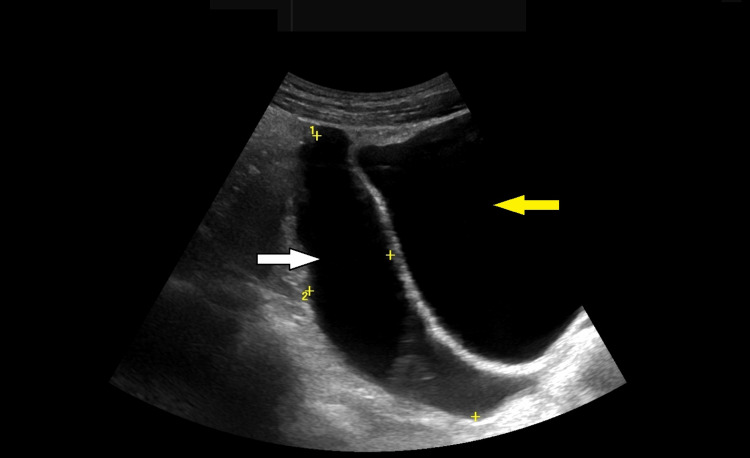
Pelvic ultrasound demonstrating a pelvic collection (denoted by the white arrow) beside the urinary bladder (denoted by the yellow arrow)

He was admitted overnight for serial examination. Following a consultant review the following morning, his abdomen was minimally tender. Appendicitis was felt to be unlikely, and an ultrasound scan of the abdomen was arranged. The appendix could not be visualised; however, a large volume of fluid was noted in the pelvis, as demonstrated in Figure 1.

**Figure 2:**
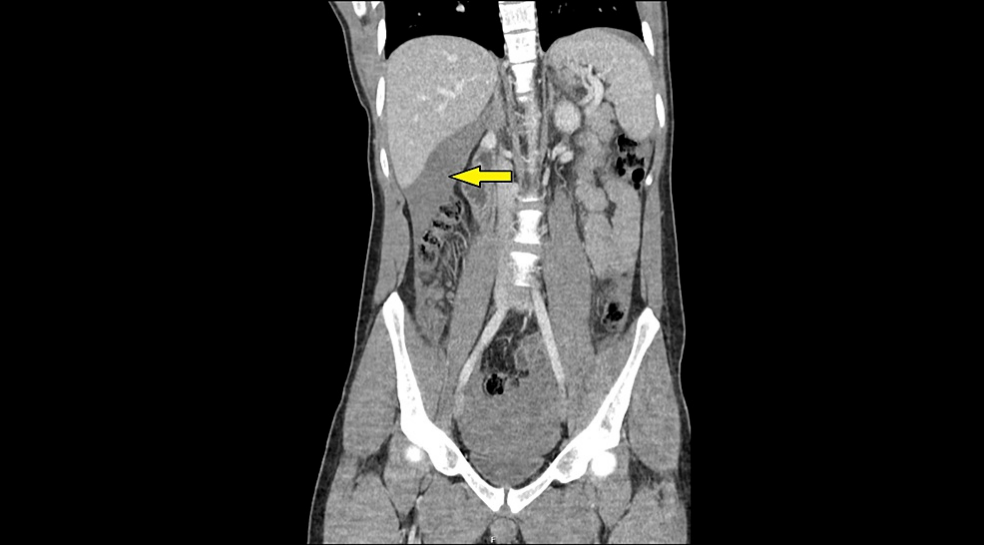
A coronal CT image of the abdomen and pelvis demonstrating high-density free fluid in the right paracolic gutter (denoted by a yellow arrow)

**Figure 3:**
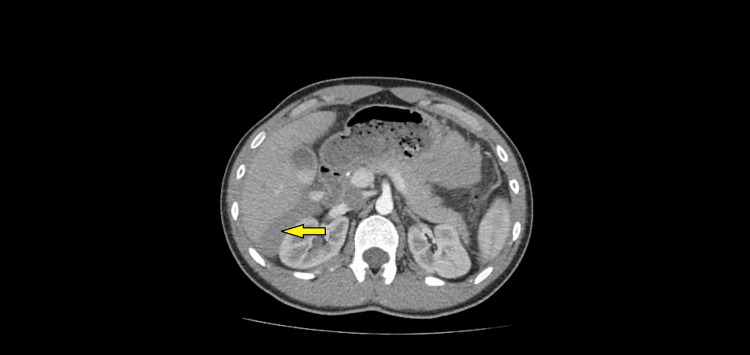
An axial CT image of the abdomen demonstrating high-density fluid, on the right side, in Morrison's pouch (denoted by a yellow arrow)

**Figure 4:**
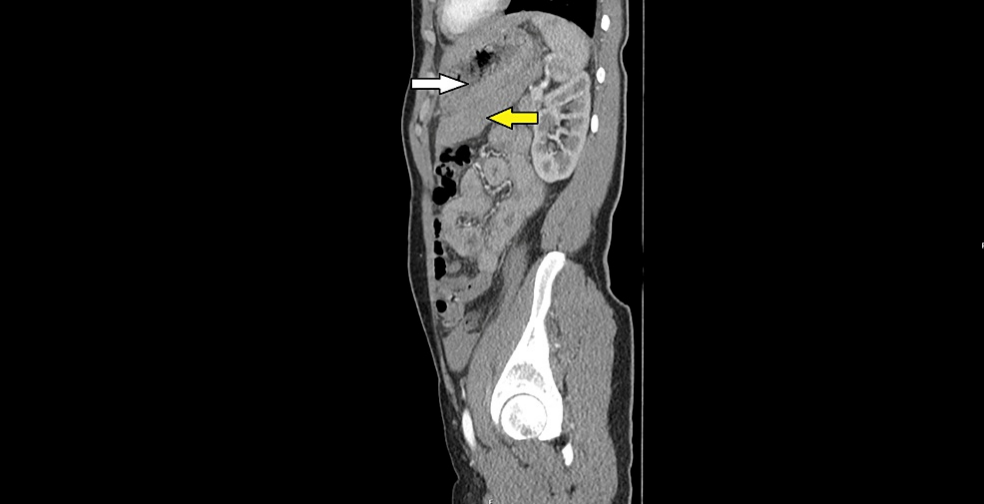
A sagittal CT image of the abdomen and pelvis. The yellow arrow highlights a high-density fluid collection related to the posterior stomach wall, which is denoted by the white arrow

Within the right lower quadrant, the fluid collection measured 13.5 x 6 cm; on the left side, it measured 7 x 5.2 cm. Repeated blood tests demonstrated Hb had fallen to 103 g/dL whilst the WCC had normalised, and the CRP had increased to 13 mg/dL. In view of these investigation findings, an urgent computed tomography (CT) scan of the patient’s abdomen and pelvis, with contrast, was arranged. This demonstrated a moderate volume of high-density fluid within the abdomen and pelvis (Figures 2-4). However, no source was identified, and a haemorrhagic cause was suggested.

The appendix was visualised and was normal. Further haematological and biochemical analysis of the patient’s blood demonstrated a consecutive fall in Hb to 93 g/dL. Additional assessment was performed with a CT angiogram of the abdomen and pelvis. This demonstrated no change in the volume of the intra-abdominal fluid, with no active bleeding.

The patient remained clinically stable throughout his admission with improvement in his symptoms. A diagnosis of ISIH was made. A conservative management approach was implemented along with intravenous tranexamic acid for a total of three doses. A senior member of the surgical team examined him daily along with daily monitoring of his haematological and biochemical markers. He continued to demonstrate clinical improvement along with recovery of his Hb and platelet levels and normalisation of his WCC. He was discharged following a six-day admission.

He was reviewed one week later; his symptoms had resolved. His blood results had returned to normal ranges and no further follow-up or imaging was required.

## Discussion

ISIH was first reported in 1909 by Barber; he described a case where significant intra-abdominal bleeding was noted [4,5]. It was later classified as abdominal apoplexy in 1931 by Green and Powers. They reported several cases of ISIH with the presenting symptom of the sudden onset of severe abdominal pain primarily in the epigastric region [6,7]. They stated that ISIH is often mistaken for a perforated peptic ulcer or acute pancreatitis. Since then cases have been reported with variable presentations from nonspecific abdominal pain associated with vomiting and anorexia to severe abdominal pain with shock and cardiovascular collapse [3,7]. Typically, a peritonitic and rigid abdomen was noted on clinical examination [6].

The condition has been shown to be three times more common in men than women and is usually seen between the ages of 40 and 60 years [1,5,8]. In these cases, arteriosclerosis and hypertension are believed to be predisposing aetiological factors [2,5,9]. Consequently, when blood pressure rises, in the presence of a weakened tunica media, a rupture occurs [3]. In our case, the patient lacked these risk factors. Methylphenidate hydrochloride has been shown to increase heart rate and blood pressure in children and adolescents; however, our patient was normotensive at presentation [10]. Congenital vessel wall malformations have been proposed to be likely causes of ISIH in younger patients [5].

The variable presentation is due to the variable nature of the haemoperitoneum [2,5]. Initially, the abdominal pain may range from mild to severe, which correlates with how rapidly the blood accumulates as well as the volume within the peritoneum [2,5,8]. Subsequently, a latent phase lasting hours to days occurs where the patient has no symptoms of the condition [5,8]. There may be a following terminal phase where increasing symptoms and signs of hypovolaemic shock are noted [2,5].

CT imaging, particularly CT angiography, has been documented to be the most effective investigation to aid in diagnosis [2,5,10]. Where the bleeding is accurately identified, percutaneous transcatheter embolization can be considered if the patient is haemodynamically stable [2,8]. Frequently, imaging does not identify the source of bleeding, as demonstrated in this case [2]. In an unstable patient, a focused assessment with sonography in trauma (FAST) scan in the emergency department may detect intra-abdominal haemorrhage [8,11].

Many cases have only been diagnosed during the surgical intervention such as laparoscopy or laparotomy [3,5,9,11]. Resuscitation and urgent surgical intervention, to identify and ligate the bleeding vessel, is the optimal management of unstable patients where ISIH is suspected [2,8]. Ruptures of the middle colic artery, left gastric artery, superior mesenteric artery and splenic artery have been identified as sources of bleeding [2,3,5]. Haematomas within the mesentery have also been found during operative management [11]. However, in approximately 40% of cases, the source of bleeding is also not identified at surgery [3,9,11]. Moreover, it has been proposed that without a surgical intervention, mortality approaches 100% [2,5,11]. In cases of non-therapeutic laparotomy, mortality between 40% and 66% has been reported [8]. With surgical intervention, identification of the bleeding source and ligation of the vessel, mortality drops below 10% [8,11].

In this case, the patient’s symptoms and clinical examination quickly and significantly improved on the implementation of conservative management. He remained haemodynamically stable throughout a substantial period of observation. Therefore, an exploratory operative intervention was not required, nor was it in the patient’s best interest at such a young age. The risks and potential post-operative complications significantly outweighed the benefits of the intervention.

Thus, a conservative management approach is safe in young, stable patients [3]. Careful monitoring through clinical examination, vital signs and blood tests is an effective approach in managing such patients.

## Conclusions

Early identification of ISIH requires a high index of clinical suspicion due to its rarity and ability to mimic other causes of an acute abdomen. Early investigation with CT imaging is likely to identify intra-abdominal haemorrhage as the cause of the patient’s symptoms. Identifying the source of bleeding may not be possible. Unstable patients require an immediate operative intervention to attempt to identify and manage the bleeding source. However, a careful clinical evaluation of examination findings, trends within vital signs such as blood pressure and assessment of Hb changes can enable a conservative management approach to be adopted in stable patients.
